# siRNA-based targeting of antiapoptotic genes can reverse chemoresistance in P-glycoprotein expressing chondrosarcoma cells

**DOI:** 10.1186/1476-4598-8-28

**Published:** 2009-05-15

**Authors:** Dae Won Kim, Kyung-Ok Kim, Mike J Shin, Jung Hee Ha, Sung Wook Seo, Jay Yang, Francis Y Lee

**Affiliations:** 1 Department of Orthopaedic Surgery, The Center for Orthopaedic Research, Columbia University, New York, NY 10032, USA; 2 Department of Anesthesiology, Columbia University, 630 West 168th Street New York, NY 10032, USA

## Abstract

**Background:**

High expression of P-glycoprotein is one of the well-known mechanisms of chemoresistance in chondrosarcomas. However, the role of antiapoptotic proteins, a common mechanism responsible for chemoresistance in other tumors, has not been well studied in chondrosarcomas. We examined the importance of P-glycoprotein and antiapoptotic proteins in the chemoresistance to doxorubicin of two Grade II chondrosarcoma cell lines, JJ012 and SW1353.

**Results:**

We confirmed that both chondrosarcoma cell types expressed P-glycoprotein and antiapoptotic proteins (Bcl-2, Bcl-xL and XIAP). siRNA knockdown as well as pharmacologic inhibitors of cell survival proteins (Bcl-2, Bcl-xL and XIAP) enhanced apoptosis of chemoresistant chondrosarcoma cells by up to 5.5 fold at 0.1 μmol and 5.5 fold at 1 μmol doxorubicin. These chemosensitizing effects were comparable to those of P-glycoprotein inhibition by siRNA or pharmacologic inhibitor.

**Conclusion:**

These findings suggest that antiapoptotic proteins play a significant role in the chemoresistance of chondrosarcoma cells independent of P-glycoprotein. Based on the results, a new siRNA-based therapeutic strategy targeting antiapoptotic genes can be designed to overcome the chemoresistance of chondrosarcomas which is often conferred by P-glycoprotein.

## Background

Chondrosarcoma is the second most common sarcoma arising in bones and the main treatment is surgical resection with a wide margin. However, there is no effective therapeutic option for metastatic chondrosarcoma patients since chondrosarcoma is resistant to both chemotherapy and radiation therapy [1,2]. Therefore, it is necessary to explore new therapeutic approaches for metastatic and surgically unresectable chondrosarcoma cases. P-glycoprotein, a product of multidrug resistant gene 1, and antiapoptotic protein overexpression are two common mechanisms of chemoresistance in tumor cells. It has already been reported that chondrosarcoma cells highly express P-glycoprotein and antiapoptotic proteins (Bcl-2, Bcl-xL, XIAP) [3-6]. The role of P-glycoprotein in drug efflux has been identified as one of the mechanisms for chemoresistance in human chondrosarcoma cells [3,7], while the function of antiapoptotic genes in chemoresistance has not been elucidated.

P-glycoprotein is a transmembrane ATP-dependent pump that transports drugs out of cells as protection against toxins. Tumor cells exposed to a single cytotoxic drug are resistant to structurally and functionally unrelated drugs, and P-glycoprotein is largely responsible for this multidrug resistance (MDR) [8,9]. MDR resulting from the overexpression of P-glycoprotein has been reported in different types of soft tissue sarcomas (eg, malignant fibrous histiocytoma, liposarcoma, leiomyosarcoma, Ewing's sarcoma) and hematologic malignancies (eg, multiple myeloma, acute myeloid or lymphoblastic leukemia) [10,11]. In addition to drug transportation, P-glycoprotein overexpressing cells exhibit abrogation of mitochondrial cytochrome c release and caspase-3 activation, which may be dependent on Bcl-xL overexpression [12]. Bcl-xL, one of the well-known antiapoptotic Bcl-2 family members, controls apoptosis by blocking the release of cytochrome c from the mitochondria. Furthermore, the activation of caspases, the effector molecules of apoptosis, is dependent on this cytochrome c release. It has been reported that the inhibition of apoptosis can lead to tumorigenesis and resistance to chemotherapy and radiotherapy in carcinomas [13,14]. Although the role of antiapoptotic proteins in the chemoresistance of chondrosarcoma is not well understood, the overexpression of antiapoptotic proteins (Bcl-2, Bcl-xL, XIAP) is one of the mechanisms of radiation resistance in chondrosarcoma cells [4]. Since chemotherapeutic agents and radiation therapy both induce apoptotic cell death [15,16], antiapoptotic proteins may contribute to chemoresistance, as well. Several studies have suggested that antiapoptotic proteins have a major role in chemoresistance [17,18]. Chondrosarcoma cells with MDR properties conferred by membrane-bound P-glycoprotein still have a significant amount of cytoplasmic levels of doxorubicin remaining after doxorubicin treatment and washout, which further supports the involvement of antiapoptotic proteins in chemoresistance [7].

Based on these findings, we hypothesize (1) antiapoptotic proteins mediate chemoresistance in chondrosarcoma cells and (2) the knockdown of these proteins, as well as P-glycoprotein, would enhance chemosensitivity to the doxorubicin remaining in the cells.

## Results

### Chondrosarcoma cells are resistant to chemotherapy

**Figure 1:**
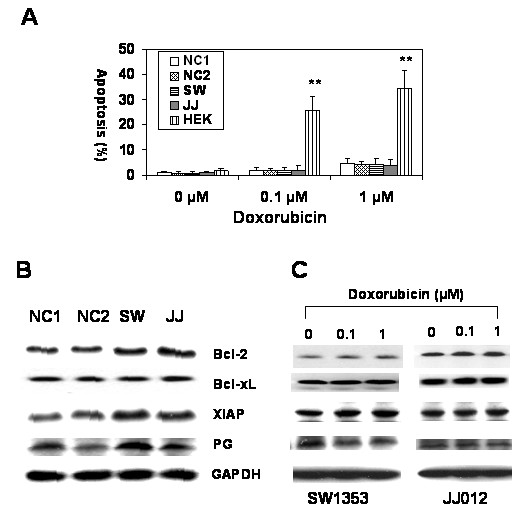
**Doxorubicin resistance and expression of antiapoptotic protein and P-glycoprotein in chondrosarcoma cells**. (A) Detection of apoptosis by flow cytometry after doxorubicin treatment. Two normal articular chondrocytes (NC1 and NC2) and two chondrosarcoma cells (SW: SW1353 and JJ: JJ012) showed chemoresistance when compared to human embryonic kidney cells (HEK). (B) Immunoblotting anti-apoptotic proteins (Bcl-2, Bcl-xL, and XIAP) and P-glycoprotein. Bcl-2, Bcl-xL, XIAP and P-glycoprotein expressions were verified in two normal chondrocytes (NC1 and NC2) and two chondrosarcoma cells (SW: SW1353, JJ: JJ012). (C) Immunoblotting anti-apoptotic proteins (Bcl-2, Bcl-xL, and XIAP) and P-glycoprotein in two chondrosarcoma cells after doxorubicin treatment. Bcl-2, Bcl-xL, XIAP and P-glycoprotein expressions were not changed significantly after doxorubicin treatment.

In order to verify the chemoresistance of chondrosarcoma cells, we treated well-known human grade II chondrosarcoma cells, SW1353 and JJ012 [19-21], with doxorubicin *in vitro*. Doxorubicin treatment did not increase apoptosis in chondrosarcoma cells while human embryonic kidney (HEK) cells were undergoing robust apoptosis (Figure 1A). Normal chondrocyte cells also exhibited chemoresistance, suggesting that chondrocytes, in general, are chemoresistant. To confirm P-glycoprotein and antiapoptotic protein expression as a possible mechanism of chemoresistance in chondrosarcoma, we measured P-glycoprotein, Bcl-2, Bcl-xL and XIAP expression by immunoblotting (Figure 1B). Normal chondrocytes and chondrosarcoma cells express all these proteins, suggesting that both P-glycoprotein and antiapoptotic proteins may contribute to doxorubicin resistance.

To investigate the effect of doxorubicin, we measured protein expression after doxorubicin treatment (Figure 1C). Doxorubicin treatment did not significantly change expression levels of P-glycoprotein and antiapoptotic proteins in both chondrosarcoma cell types.

### P-glycoprotein is expressed on the cell surface and expels doxorubicin from the cells

**Figure 2:**
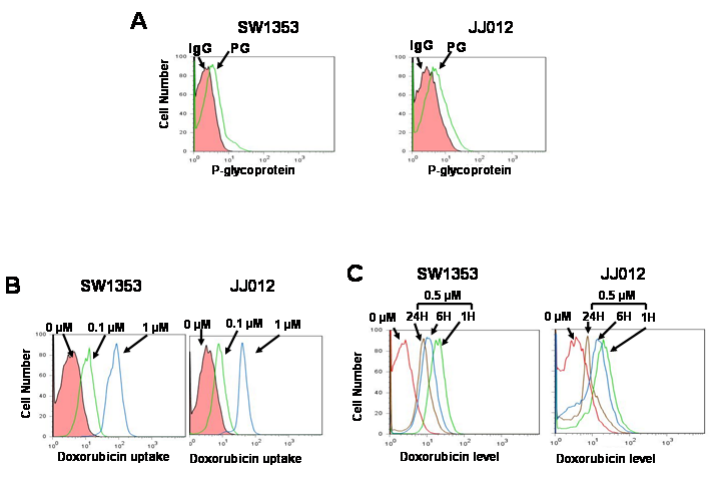
**Cell surface expression of P-glycoprotein and doxorubicin uptake and efflux in chondrosarcoma cells**. (A) Cell surface P-glycoprotein expression was detected by flow cytometric analysis. Cell surface P-glycoprotein staining was positive in two chondrosarcoma cells. (IgG: isotype IgG control, PG: P-glycoprotein antibody) (B) Doxorubicin uptake was measured in two chondrosarcoma cells. Doxorubicin uptake increased as doxorubicin concentration was increased in the two chondrosarcoma cell lines. (C) Functional assay of P-glycoprotein was performed after doxorubicin washout. Doxorubicin levels were measured at 1, 6 and 24 hours after washout after 1 hour treatment. Doxorubicin levels decreased in a time dependant manner.

Membrane-bound P-glycoprotein expression and doxorubicin uptake were measured by flowcytometry. P-glycoprotein was present on the cell surface of both chondrosarcoma cell types (Figure 2A). Doxorubicin has inherent autofluorescent (excitation wavelength: 480 nm, emission wavelength: 580 nm) and intracellular doxorubicin uptake was measured. Our results show that as doxorubicin dose increased, the number of fluorescent cells increased (Figure 2B). To examine the functional activity of P-glycoprotein, we measured doxorubicin levels in chondrosarcoma cells after doxorubicin washout. Doxorubicin levels decreased in cells in a time dependant manner (Figure 2C). However, a substantial amount of doxorubicin still remained 24 hours after doxorubicin washout (Figure 2C). These data suggest that P-glycoprotein may not be the only responsible mechanism for chemoresistance by chondrosarcoma cells.

### Pharmacologic inhibitors of antiapoptotic protein and P-glycoprotein enhance doxorubicin sensitivity

**Figure 3:**
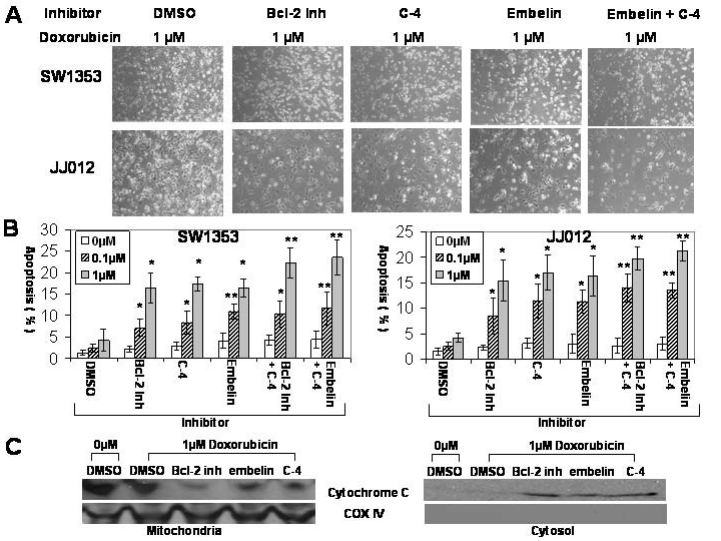
**Apoptosis after inhibitor and doxorubicin treatment**. (A) Microscopic pictures after inhibitor and doxorubicin treatment are shown. Chondrosarcoma cells treated with 10 μM of Bcl-2 inhibitor, 10 μM of C-4 (P-glycoprotein inhibitor) or 10 μM of embelin (XIAP inhibitor) showed more apoptotic cells than control groups after doxorubicin treatment. (Bcl-2 inh: Bcl-2 inhibitor, original magnification ×100). (B) The effect of inhibitors on chemosensitivity in chondrosarcoma cells is shown. Inhibitor treatment enhanced chemosensitivity. Inhibitor treated chondrosarcoma cells demonstrated increased apoptosis after doxorubicin treatment in comparison to the control groups (up to 4.5 fold increase). Combination treatment of inhibitors (Bcl-2 inh + C-4 and embelin + C-4) showed significant increase in apoptosis. (Bcl-2 inh: Bcl-2 inhibitor, *: p < 0.05 and **: p < 0.01) (C) Immunoblotting demonstrates cytochrome c release from mitochondria to cytosol after treatment with doxorubicin and inhibitors. Cytochrome oxidase IV (COX IV) serves as a marker for mitochondria.

We showed that P-glycoprotein does not completely eliminate doxorubicin from chondrosarcoma cells. We hypothesize that antiapoptotic proteins have a critical role in chemoresistance since a significant amount of doxorubicin remains within chondrosarcoma cells. To test our hypothesis, we treated cells with inhibitors of antiapoptotic proteins and P-glycoprotein in the presence or absence of doxorubicin. First, we performed a dose-response experiment using 3 different doses of pharmacologic inhibitors based on previously published reports in order to find an optimal concentration that is least cytotoxic [22-24]. There were no significant changes in the rate of apoptosis with 10 μM of Bcl-2 inhibitor (2.36 ± 0.67%), 10 μM of C-4 (3.19 ± 0.91%) or 10 μM of embelin (3.53 ± 1.75%) when compared with the DMSO treated control group (1.53 ± 0.74%). Based on these data, chondrosarcoma cells were treated with 10 μM of Bcl-2 inhibitor, 10 μM of C-4 (P-glycoprotein inhibitor) or 10 μM of embelin (XIAP inhibitor) and two different concentrations of doxorubicin (0.1 μM and 1 μM). Apoptosis rates were measured to investigate whether or not inhibition of antiapoptotic proteins or P-glycoprotein would enhance doxorubicin sensitivity in chondrosarcoma cells. While the DMSO treated chondrosarcoma cells exhibited chemoresistance (SW1353; 2.42 ± 0.96% at 0.1 μM, 4.24 ± 2.53% at 1 μM. JJ012; 2.5 ± 0.76% at 0.1 μM, 4.26 ± 0.9% at 1 μM), inhibition of Bcl-2, XIAP and P-glycoprotein enhanced the chemosensitivity by up to 4.5 fold (C-4 treated JJ012: 11.46 ± 3.37%, p < 0.05) at 0.1 μM and 4 fold (C-4 treated SW1353: 17.36 ± 1.58%, p < 0.05) at 1 μM in comparison to each control group on flowcytometric examination (Figures 3A and 3B). In addition, the combination of inhibitors for antiapoptotic proteins and P-glycoprotein enhanced the chemosensitivity by up to 5.5 fold (Bcl-2 inhibitor+C-4 treated JJ012: 13.93 ± 2.76%, p < 0.01) at 0.1 μM, 5.5 fold (embelin+C-4 treated SW1353:23.51 ± 3.50%, p < 0.01) at 1 μM. The increase in the chemosensitizing effect by combinatorial use of antiapoptotic protein and P-glycoprotein inhibitors was maximal when P-glycoprotein (C-4) and XIAP (embelin) were inhibited simultaneously in doxorubicin treatment (Figure 3B).

To examine the mechanism of doxorubicin and inhibitor induced cell death in chondrosarcoma cells, we obtained cytosolic and mitochondrial fractions and measured cytochrome c expression after treatment of doxorubicin in the presence or absence of inhibitors. Doxorubicin treatment with inhibitors of antiapoptotic protein or P-glycoprotein induced cytochrome c release from mitochondria to cytosol (Figure 3C). However, doxorubicin treatment alone did not trigger mitochondrial cytochrome c release significantly. These data suggest inhibition of antiapoptosis proteins and P-glycoproteins enhance doxorubicin sensitivity by the cytochrome c release mechanism.

**Figure 4:**
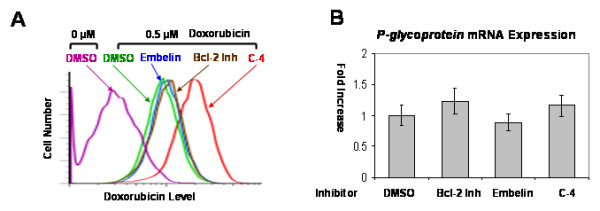
**Function and mRNA expression of P-glycoprotein after inhibitor treatment**. (A) Doxorubicin efflux was measured after inhibitor treatment. Only C-4 (P-glycoprotein inhibitor) treatment inhibited doxorubicin efflux significantly. (Bcl-2 inh: Bcl-2 inhibitor) (B) mRNA expression of *P-glycoprotein *was measured by RT-PCR. All inhibitors did not change mRNA expression of *P-glycoprotein*. mRNA expression of *P-glycoprotein *of each inhibitor treatment was divided by mRNA expression of DMSO control. (Bcl-2 inh: Bcl-2 inhibitor).

There is a possibility that antiapoptotic protein inhibitors treatment may change the function or the expression of P-glycoprotein instead of the direct effect on antiapoptotic proteins in P-glycoprotein expressing chondrosarcoma cells. To investigate the possibility, we performed doxorubicin efflux assay and measured *P-glycoprotein *mRNA expression by RT-PCR after treatment of antiapoptotic protein inhibitors. Antiapoptotic inhibitors treatment (Bcl-2 inhibitor, embelin) changed P-glycoprotein function, but it was not significant (Figure 4A). Only C-4 treatment (P-glycoprotein inhibitor) inhibited doxorubicin efflux significantly compared with DMSO control (Figure 4A). None of the inhibitors treatment changed mRNA expression of *P-glycoprotein *(Figure 4B). These data suggest antiapoptotic proteins and P-glycoprotein may be independent chemoresistant mechanisms in chondrosarcoma cells.

### Antiapoptotic and *P-glycoprotein *gene silencing enhances doxorubicin sensitivity

**Figure 5:**
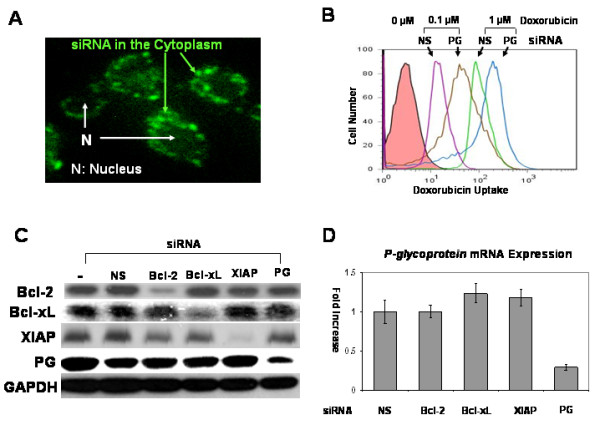
**siRNA gene silencing**. (A) A fluorescent microscopy picture demonstrates intracellular localization of siRNA tagged with green fluorescent protein (GFP). Most GFP tagged siRNA was seen in the cytoplasm while the nuclei (N) lack green fluorescence. (B) Doxorubicin level was measured after *P-glycoprotein *siRNA treatment. *P-glycoprotein *siRNA treatment increased doxorubicin level at 0.1 and 1 μM in comparison to Non-Silencing control siRNA. (NS: Non-Silencing siRNA, PG: *P-glycoprotein *siRNA) (C) Immunoblotting demonstrates decreased expression of Bcl-2, Bcl-xL, XIAP and P-glycoprotein by siRNA. (-: carrier only, NS: Nonsilencing siRNA, PG: P-glycoprotein) (D) Targeting antiapoptotic gene (*Bcl-2*, *Bcl-xL *and *XIAP*) did not change mRNA expression of *P-glycoprotein*. Only *P-glycoprotein *siRNA treatment decreased mRNA expression of *P-glycoprotein*. mRNA expression of *P-glycoprotein *of each siRNA treatment was divided by mRNA expression of nonsilencing control. (NS: Nonsilencing siRNA, PG: P-glycoprotein).

**Figure 6:**
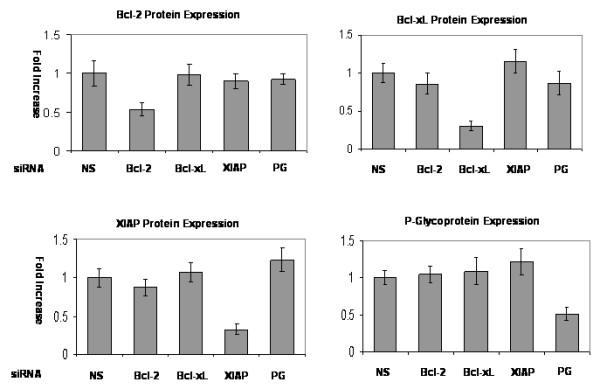
**Quantification of protein expression after siRNA gene silencing**. Protein expression was quantified by ImageJ software after siRNA gene silencing. Only targeted gene expression changed significantly compared with gene expression of non-silencing control. Non-target gene expression did not change significantly. (NS: Nonsilencing siRNA, PG: P-glycoprotein).

We also tested our hypothesis that both antiapoptotic proteins and P-glycoprotein play an important role in the chemoresistance of chonodrosarcoma cells using siRNA. First, we examined intracellular uptake of siRNA which has a fluorescent tag. Chondrosarcoma cells showed fluorescence in the cytoplasm 24 hours after transfection with fluorescence protein tagged siRNA (Figure 5A). We examined the P-glycoprotein drug-exporting function after *P-glycoprotein *gene silencing. P-glycoprotein knockdown decreased doxorubicin efflux significantly (Figure 5B). Next, immunoblotting was used to confirm siRNA's target gene knockdown effect (Figure 5C). Target protein expression of each siRNA was significantly decreased in comparison to the control groups. To determine whether targeting antiapoptotic genes may affect the expression of P-glycoprotein or vice versa, protein expression was measured by quantifying the density of immunoblot bands adjusted to GAPDH using ImageJ software and mRNA expression of *P-glycoprotein *was measured by RT-PCR after siRNA treatment. Targeting any of the antiapoptic genes did not change mRNA and protein expression of *P-glycoprotein *gene significantly compared with non-silencing control (Figure 5D and Figure 6). Targeting the *P-glycoprotein *gene did not change any of the antiapoptotic proteins expression, either (Figure 6). These data support our inhibitors experiment finding (Figure 4A, 4B), which suggests antiapoptotic proteins and P-glycoprotein are independent doxorubicin resistant mechanisms in chondrosarcoma cells.

**Figure 7:**
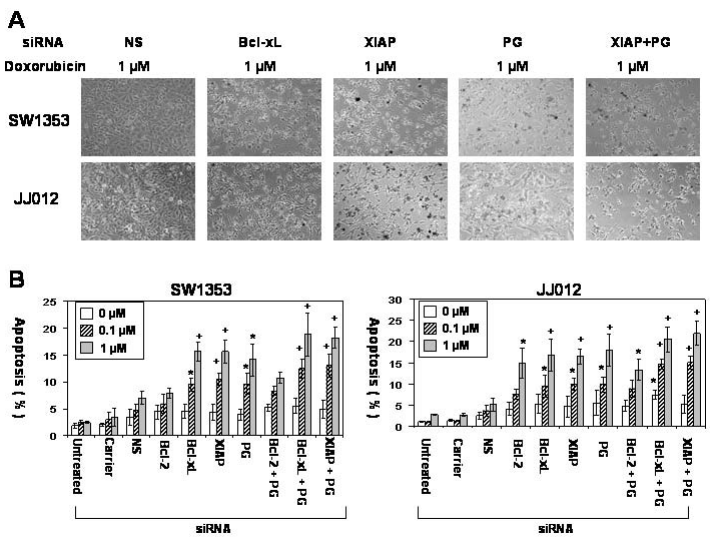
**Apoptosis after siRNA and doxorubicin treatment**. (A) Microscopic pictures after siRNA and doxorubicin treatment are shown. Chondrosarcoma cells treated with siRNAs showed more apoptotic cells than control groups after doxorubicin treatment (NS: nonsilencing siRNA, PG: *P-glycoprotein*, original magnification ×100). (B) The effect of gene silencing on chemosensitivity in chondrosarcoma cells is shown. siRNA treatment enhanced chemosensitivity. siRNA treated chondrosarcoma cells demonstrated increased apoptosis after doxorubicin treatment in comparison to the control groups (up to 3.3 fold increase). Dual gene silencing (*Bcl-xL*+PG and *XIAP *+PG siRNA) also increased apoptosis significantly. (NS: nonsilencing siRNA, PG: *P-glycoprotein*, *: p < 0.05, +: p < 0.01, Nonsilencing siRNA treated group was used as a control for p-value.)

We calculated the following target gene knockdown efficiency with 1 μg/ml of siRNA (SW1353; *Bcl-2*: 39 ± 5.1%, *Bcl-xL*: 72 ± 10.3%, *XIAP*: 66 ± 12.2%, *P-glycoprotein*: 47 ± 6.8%. JJ012; *Bcl-2*: 57 ± 10.2%, *Bcl-xL*: 75 ± 11.7%, *XIAP*: 70 ± 9.6%, *P-glycoprotein*: 62 ± 9.9%). Apoptosis rates were measured with annexin V after chondrosarcoma cells were treated with 1 μg/ml of siRNA and two different concentrations of doxorubicin (0.1 μM and 1 μM). While the untreated chondrosarcoma cells exhibited chemoresistance, *Bcl-2*, *Bcl-xL *and *XIAP *gene silencing enhanced chemosensitivity by up to 2.7 fold (*XIAP *siRNA treated JJ012: 9.97 ± 1.47%, p < 0.05) at 0.1 μM and 3.3 fold (*Bcl-xL *siRNA treated JJ012: 16.90 ± 3.84%, p < 0.01) at 1 μM in comparison to each nonsilencing control group (JJ012: 3.63 ± 1.27% at 0.1 μM, 5.12 ± 1.49% at 1 μM) on flow cytometric examination (Figure 7A and 7B). *P-glycoprotein *gene silencing also enhanced chemosensitivity significantly at 0.1 μM (SW1353: 9.65 ± 1.98%, JJ012: 9.83 ± 1.72%, p < 0.05) and 1 μM (SW1353: 14.12 ± 3.03%, JJ012: 17.99 ± 3.77%, p < 0.05) in comparison to each nonsilencing control group. Additionally, dual gene silencing of both the antiapoptotic gene and *P-glycoprotein *gene enhanced chemosensitivity by up to 4.1 fold (*XIAP*+PG siRNA treated JJ012: 15.04 ± 1.60%, p < 0.01) at 0.1 μM, and 4.2 fold (*XIAP*+PG siRNA treated JJ012: 21.96 ± 2.79%, p < 0.01) at 1 μM. Interestingly, antiapoptotic or *P-glycoprotein *gene silencing without doxorubicin treatment caused a very modest induction of apoptosis (Figure 7B). Overall, the use of target protein specific siRNA and doxorubicin increased chemosensitivity by 4 to 14 folds in comparison to untreated cells.

### Antiapoptotic or P-glycoprotein gene silencing with chemotherapy decreases cell survival and proliferation

**Figure 8:**
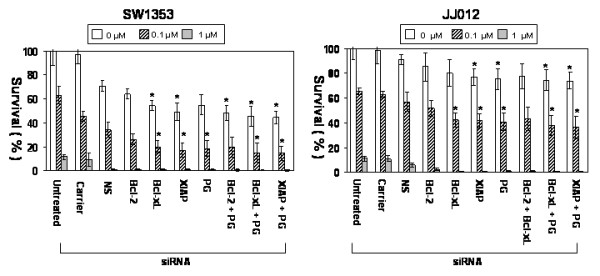
**Clonogenic survival after siRNA and doxorubicin treatment**. Clonogenic survival after chemotherapy and siRNA is shown. siRNA treatment with chemotherapy decreases colony formation by two times. Combinatorial siRNA treatments (*Bcl-xl *+ PG or *XIAP *+ PG siRNA) inhibited colony formation effectively after doxorubicin treatment. (PG: *P-glycoprotein*, *: p < 0.05).

Tumor recurrence is a prognostic factor which negatively affects clinical outcome following radiation or chemotherapy. We hypothesize that anti-apoptotic or P-glycoprotein gene silencing in combination with chemotherapy decreases both survival and proliferation of chondrosarcoma cells. Clonogenic cell survival assays were conducted and showed that chondrosarcoma cells still have the ability to proliferate and form multiple colonies after doxorubicin treatment (Figure 8). When antiapoptotic or *P-glycoprotein *genes were silenced in cells that underwent chemotherapy, the number of colonies decreased significantly (p < 0.05). Antiapoptotic gene silencing decreased colony formation by up to three fold (p < 0.05) at 0.1 μM, in comparison to the control group. At the highest dose of doxorubicin (1 μM), there was no significant colony formation in any siRNA treated group.

## Discussion

There is no effective treatment for metastatic or unresectable chondrosarcomas due to the chemo- and radioresistant properties of such cancers. In this regard, a better understanding of the molecular mechanisms responsible for chemo- and radioresistance is necessary to develop novel therapeutic strategies. Failure of chemotherapy results from both patient and tumor variances. The host factors include poor absorption, rapid metabolism and insufficient drug delivery to the tumor site, while the tumor factors include the loss of cell surface receptors or drug transport proteins, specific metabolism of chemotherapy drugs, mutation of the specific target of drugs, and the increase in drug efflux [25]. Drug efflux has been studied extensively, since it has the most relevance to multidrug resistance. Consequently, it is believed that the major mechanism of multidrug resistance in tumor cells is P-glycoprotein expression [26]. Several *in vitro *studies have already reported that most chondrosarcoma cells express P-glycoprotein to confer MDR [5,7]. Our experiments have confirmed this as previously reported by other groups (Figure 1B). Doxorubicin resistant normal cartilage and chondrosarcoma cells express high levels of antiapoptotic proteins as well as P-glycoprotein, suggesting that both P-glycoprotein and antiapoptotic proteins may contribute to doxorubicin resistance.

P-glycoprotein is located on the plasma membrane which removes cytotoxic drugs from the cell. It is believed to be the main mechanism of chemoresistance in P-glycoprotein expressing tumors regardless of antiapoptotic gene expression since cytotoxic drugs were thought to be removed before antiapoptotic proteins would work. However, our data reveals that doxorubicin uptake increases with higher doses in P-glycoprotein expressing chondrosarcoma cells (Figure 2B) and antiapoptotic protein inhibition and gene silencing enhanced doxorubicin sensitivity independent of P-glycoprotein (Figures 3B and 7B). Additionally, significant amounts of doxorubicin still remained in chondrosarcoma cells 24 hours after doxorubicin washout (Figure 2C).

**Figure 9:**
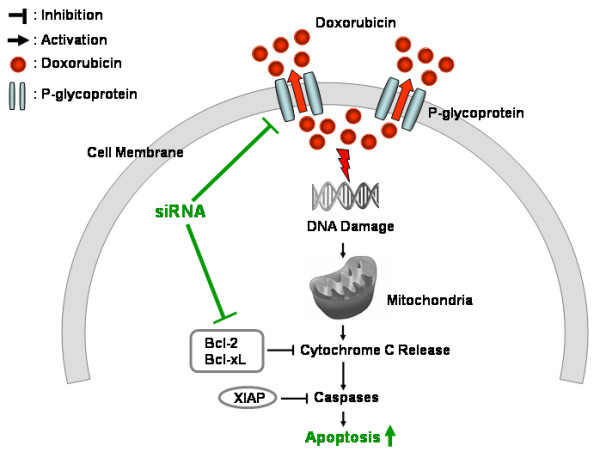
**Doxorubicin resistance mechanisms in chondrosarcoma cells**. A schematic diagram demonstrates a molecular pathway of apoptosis that is triggered by doxorubicin. Doxorubicin damages DNA. This DNA damage induces cytochorome C release in mitochondria which activates caspases, the effectors of apoptosis. P-glycoprotein inhibits doxorubicin effect by drug efflux. Bcl-2 and Bcl-xL block cytochrome c release. XIAP inhibits caspase activation.

Our results suggest that antiapoptotic proteins play a role in the chemoresistance of chondrosarcoma cells by enhancing cell survival in addition to P-glycoprotein. These antiapoptotic proteins and P-glycoprotein are two independent chemoresistance mechanisms in chondrosarcoma cells since targeting antiapoptotic proteins did not have any effect on the activity, mRNA expression level and protein expression level of P-glycoprotein (Figure 4A, B, 5D and 6) and targeting P-glycoprotein did not change the expression level of antiapoptotic proteins, either (Figure 6). The two independent mechanisms are closely related in terms of a cell death pathway by blocking cytochrome c release for doxorubicin resistance in chondrosarcoma cells (Figure 3C and 9). We also found targeting both P-glycoprotein and antiapoptotic protein with doxorubicin treatment showed additive effect rather than synergistic effect (Figure 3B and 7B). This may be due to the fact that apoptosis which results from targeting antiapoptotic proteins, induces the cleavage of P-glycoprotein [27] which may weaken the effect of targeting P-glycoprotein.

Among the antiapoptotic genes screened, the knockdown of *Bcl-xL *and *XIAP *enhanced doxorubicin sensitivity as effectively as *P-glycoprotein *and the combined knockdown of *Bcl-xL *with *P-glycoprotein *and *XIAP *with *P-glycoprotein *were significantly effective in our dual gene silencing group (Figure 7A and 7B). This may be explained by the fact that Bcl-xL and XIAP overexpression in P-glycoprotein expressing tumor cells has been associated with a much stronger resistance to treatment and a worse prognosis [12,28]. Moreover, apoptosis itself induces the cleavage of P-glycoprotein, which may enhance apoptotic sensitivity [27].

Yet, the knockdown of *Bcl-2 *did not induce significant apoptosis in SW1353 chondrosarcoma cells (Figure 7B) while Bcl-2 inhibitor treatment enhanced doxorubicin sensitivity in SW1353 cells (Figure 3B). This may be due to the low knockdown efficiency of *Bcl-2 *(SW1353; *Bcl-2*: 39 ± 5.1%, *Bcl-xL*: 72 ± 10.3%, *XIAP*: 66 ± 12.2%, *P-glycoprotein*: 47 ± 6.8%. JJ012; *Bcl-2*: 57 ± 10.2%, *Bcl-xL*: 75 ± 11.7%, *XIAP*: 70 ± 9.6%, *P-glycoprotein*: 62 ± 9.9%). In addition, Bcl-2 inhibitor inhibits Bcl-xL as well as Bcl-2 since Bcl-2 inhibitor competes with Bak BH3 peptide for binding to Bcl-2 and Bcl-xL [24]. Consequently, we could not establish its role in chemoresistance. There are several factors which can explain this discrepancy in the knockdown efficiency of each target protein. siRNA efficiency can be determined by the cell type, passage number, confluency of cells, turnover rate of proteins, and the stability of proteins [29]. Each protein has different turnover rates and stabilities which is why our siRNA knockdown efficiency is quite different for each target protein.

Although chondrosarcoma cells are resistant to doxorubicin, clonogenic survival data showed that the colony numbers of the untreated groups were decreased significantly at 0.1 and 1 μM of doxorubicin. This may be the result of a number of factors including trypsinization and the small number of cells seeded, in addition to doxorubicin's effect.

We used Grade II chondrosarcoma cells which are well characterized and have been widely used by other investigators [19-21]. Grade III chondrosarcoma cells could be a more attractive experimental model but grade III chondrosarcoma cells often lose cartilage phenotypes with more heterogeneous cellularity. Hence, grade II chondrosarcomas were the best choice for our purposes since they have a high resistance to both chemo- and radiotherapy, metastatic potential and a high recurrence rate but still maintain the cartilaginous phenotype. Although our study was limited to Grade II chondrosarcomas, the proposed therapeutic concept may be relevant to other high grade sarcomas including Grade III chondrosarcomas which overexpress P-glycoprotein and antiapoptotic proteins [3,6].

In summary, our data suggest that antiapoptotic proteins as well as P-glycoprotein confer chemoresistance to P-glycoprotein expressing chondrosarcoma cells (Figure 9). As a result, antiapoptotic gene and *P-glycoprotein *silencing via siRNA possibly could be used as a molecular adjuvant therapy for metastatic or surgically unresectable chondrosarcomas. However, additional *in vivo *studies will be necessary before applying the proposed therapeutic concept in clinical trials.

## Methods

### Chondrosarcoma and Normal Chondrocyte Cell Cultures

Two well-establised Grade II chondrosarcoma and two normal chondrocyte cell lines were used. The chondrosarcoma cell line, JJ012, was obtained from Dr. Joel A. Block (Rush Medical College, Chicago, Illinois, USA), and the other chondrosarcoma cell line, SW1353, was purchased from American Type and Culture Collection (Manassas, Maryland, USA). Two normal articular chondrocyte tissue samples were harvested from patients after obtaining IRB approval. Cartilage specimens were minced with scissors in DMEM (Invitrogen, Carlsbad, California, USA), producing a cell suspension of small tissue fragments. The suspension was pelleted by centrifugation and the tissue fragments were digested enzymatically in phosphate buffered saline (PBS) containing 1 mg/ml collagenase, 0.15 mg/ml DNAse, and 0.15 mg/ml hyaluronidase (Sigma, St. Louis, Missouri, USA) for 1 hour at 37°C. The cells were grown at 37°C in a humidified atmosphere containing 5% CO_2 _and 95% air. The culture medium consisted of 40% Dulbecco's modification of Eagle's medium, 40% MEM-α, F-12 (Invitrogen, Carlsbad, CA), 10% fetal bovine serum (Gembio, Woodland, California, USA), 100 ng/ml human insulin (Lilly, Indianapolis, Indiana, USA), 25 μg/ml ascorbic acid, and 100 nM hydrocortisone (Sigma, St. Louis, Missouri, USA). The culture medium was changed every three to four days.

### RNA Interference Targeting Anti-Apoptotic Genes and P-glycoprotein

The siRNAs targeting *Bcl-2*, *Bcl-xL*, *XIAP *and *P-glycoprotein *were obtained from Ambion (Austin, Texas, USA). Chondrosarcoma cells that were not treated with siRNAs or those treated with non-silencing siRNAs were used as negative controls. The delivery of siRNAs was verified by using FITC tagged negative control siRNAs (Qiagen, Valencia, California, USA) and X-tremeGENE siRNA transfection reagent (Roche, Branchburg, New Jersey, USA) as a carrier. Manufacturer's protocol was followed for siRNA transfection. We have reported that the optimal siRNA concentration is 1 μg/ml and gene silencing effect of siRNA is maximal on day 2 after siRNA transfection in SW1353 and JJ012 chondrosarcoma cells [4]. Therefore, transfection was carried out using 1 μg/ml of siRNA for single siRNA treatment groups and 0.5 μg/ml of each siRNA for dual treatment groups to make the total amount of siRNA administered 1 μg/ml. Doxorubicin was treated 48 hours after siRNA treatment.

### Flow Cytometry after Annexin V Staining

The culture conditions were identical for both control and experimental groups. Chondrosarcoma cells and normal chondrocytes were grown in six-well plates and treated with 0, 0.1, and 1 μM of doxorubicin for 24 hours once a confluency of 80% had been reached. Human embryonic kidney cells (HEK cells) were used as a control to verify the cytotoxic effects of doxorubicin where the dose was chosen based on peak plasma level (1–2 μg/ml or 1.7–3.4 μM) in patients receiving a standard doxorubicin treatment [30]. Another set of chondrosarcoma cells were pretreated with inhibitors including Bcl-2 inhibitor (2,9-Dimethoxy-11,12-dihydrodibenzo-diazocine 5,6-dioxide and 5,5'-Dimethoxy-2,2'-dinitrosobenzyl, Calbiochem, San Diego, California, USA), C-4 (Calbiochem, San Diego, CA) and embelin (Calbiochem, San Diego, CA) 24 hours prior to doxorubicin treatment or with siRNAs that targeted *Bcl-2*, *Bcl-xL*, *XIAP *and *P-glycoprotein *48 hours before doxorubicin treatment. Flow cytometric analysis was used to identify cells undergoing apoptosis. After trypsinizing the cells, they were stained with APC conjugated Annexin V (BD Pharmingen, San Diego, CA). Stained cells were then counted with a flow cytometer (FACS Calibur; Becton Dickinson Science, San Jose, CA).

### Doxorubicin Efflux Assay

Doxorubicin efflux assays were carried out as previously described [7]. In brief, cells were exposed with doxorubicin contained medium for 1 hour and washed with PBS two times. Then cells were incubated in doxorubicin-free medium for 1, 6 and 24 hours and harvested for measurement of autofluorescent doxorubicin (excitation wavelength: 480 nm, emission wavelength: 580 nm) with a flow cytometer.

### Clonogenic Survival Assay

The clonogenic survival assay was used to determine the capacity for cell survival and proliferation after radiation or chemotherapy [31]. After treatment with siRNA and doxorubicin, 1000 cells from each group were seeded onto 60 mm cell culture plates containing the culture medium. Fifteen days later, the cells were stained with crystal violet (Sigma, St. Louis, Missouri, USA). Colonies larger than 50 cells were counted at low magnification.

### Immunoblotting

Immunoblotting assays were conducted in order to determine the expression of P-glycoprotein and anti-apoptotic proteins by chondrosarcoma cells and the effect of gene silencing. The cells were lysed using buffer IP (10 mM Tris-HCl, pH 7.4, 150 mM NaCl, 1% Triton X-100, 0.25% Nonidet P-40, and 2 mM EDTA) supplemented with a protease inhibitor cocktail (Roche, Branchburg, New Jersey, USA). Equivalent protein extracts (10 μg) from each sample were electrophoresed in 4–20% Tris-Glycine gels (Invitrogen, Carlsbad, CA). For mitochondrial and cytosolic fraction, cells were washed with ice-cold PBS and lysis buffer A (20 mM HEPES pH7.5, 0.1% BSA, 0.1 μM PMSF, 1 mM EDTA, 1 mM DTT, 20 μg/ml leupeptin, 10 μg/ml aprotinin, 10 μg/ml pepstatin A) containing 250 mM of sucrose was added. After 10 min incubation on ice, cells were homogenized with a dounce homogenizer and centrifuged at 700 × g for 5 min at 4°C. Supernatants were centrifuged at 10,000 × g for 30 min at 4°C. The resulting supernatants were used as the cytosolic fraction and the mitochondria pellets were resuspended with lysis buffer A. Equivalent protein extracts (60 μg for mitochondrial fraction and 120 μg for cytosolic fraction) from each sample were electrophoresed in 4–20% Tris-Glycine gels (Invitrogen). The total amount of protein was quantified using the BCA assay. The protein was transferred to an Immun-Blot PVDF membrane (Bio-Rad, Hercules, California, USA) which was then incubated with Bcl-2, Bcl-xL, XIAP (Cell Signaling, Beverly, MA), α-actin (Sigma, St. Louis, Missouri, USA), P-glycoprotein (Calbiochem, San Diego, California, USA) and GAPDH antibodies (Chemicon, Temecula, California, USA). The signal strength of each immunoblot band was normalized to GAPDH and quantified using the ImageJ software.

### Quantitative Real Time RT-PCR

Total RNA was isolated from cells using an RNeasy Mini Kit (Qiagen, Valencia, California, USA) according to the manufacturer's guidelines. Single stranded cDNA was synthesized from total RNA with the SuperScript III system (Invitrogen). Real time RT-PCR for each target was performed with LightCycler FastStart DNA Master^PLUS ^SYBR Green I (Roche Diagnostics Corporation, Indianapolis, Indiana, USA) using the Smart Cycler^® ^System (Cepheid, Sunnyvale, California, USA). Primers sets used were: for GAPDH, (5'-AGAACATCATCCCTGCATCC-3') and (5'-AGTTGCTGTTGAAGTCGC-3'); for P-glycoprotein, (5'-AACAACGCATTGCCATAGCTCGTG-3') and (5'-AGTCTGCATTCTGGATGGTGGACA-3'). The thermal cycling condition consisted of pre-heating (10 minutes at 94°C) and 40 cycles of denaturation (10 seconds at 94°C), annealing (20 seconds at 60°C) and elongation (20 seconds at 72°C). Each mRNA level was normalized with the internal control GAPDH mRNA level.

### Statistical Analysis

Experiments were performed three times and in triplicate at each time. Statistical analysis was performed using the Statistical Package for the Social Sciences (SPSS) software (version 13, Chicago, Illinois, USA). Differences between each control and experimental groups were analyzed by using one-way analysis of variance between groups (ANOVA/Scheffe), and p < 0.05 was considered statistically significant.

## Competing interests

The authors declare that they have no competing interests.

## Authors' contributions

DK, JY and FL designed research. DWK, KK, MS, JHH and SWS performed molecular study. KK, JHH and SWS performed statistical analysis. DWK, JY and FL participated in data interpretation. DWK, MS and FL drafted the manuscript. All authors read and approved the final manuscript.
